# In Vitro Release of Curcumin and Resveratrol from Polymeric Systems: Films and Hydrogel

**DOI:** 10.3390/gels12070653

**Published:** 2026-07-21

**Authors:** Ana Júlia Panserini de Goes, Heloisa Januário Ribeiro de Queiroz, Lorena Trezena Sidiropoulos, Ana Lídia Piccolo Vespasiano, Gisele Mara Silva Gonçalves

**Affiliations:** 1 School of Pharmaceutical Sciences—School of Life Sciences, Pontifícia Universidade Católica de Campinas (PUC-Campinas), Campinas 13086-900, SP, Brazil; ana.jpg@puc-campinas.edu.br (A.J.P.d.G.); heloisa.jrq@gmail.com (H.J.R.d.Q.); lorena.ts@puccampinas.edu.br (L.T.S.); ana.lpv@puccampinas.edu.br (A.L.P.V.); 2 Post-Graduation Program in Health Sciences, School of Life Sciences, Pontifícia Universidade Católica de Campinas (PUC-Campinas), Campinas 13086-900, SP, Brazil

**Keywords:** polymeric films, hydrogel, resveratrol, curcumin, wound healing, in vitro drug release

## Abstract

Chronic wounds are a persistent clinical and public health challenge due to impaired tissue repair caused by sustained inflammation, oxidative stress, and cellular senescence. Natural polyphenols such as curcumin and resveratrol, alongside mesenchymal stem cell (MSC) secretome, have demonstrated complementary anti-inflammatory, antioxidant, and pro-angiogenic properties with potential for wound healing. This study reports two complementary in vitro investigations evaluating the release profiles of curcumin and resveratrol from two polymeric platforms: poly(vinyl alcohol)/sodium alginate/carboxymethylcellulose films (Study 1) and an acrylate copolymer-based hydrogel incorporating MSC secretome (Study 2). UV-Vis spectrophotometric analysis confirmed analytical selectivity with no interference from excipients. Resveratrol exhibited progressive and consistent release from the hydrogel. Curcumin compromised polymer matrix integrity and reduced resveratrol release efficiency. Also showed unsatisfactory release in both systems, attributed to its low aqueous solubility. These results support the use of resveratrol-loaded polymeric matrices as promising sustained-release platforms for bioactive wound dressings and highlight the need for nanoencapsulation strategies to improve curcumin bioavailability.

## 1. Introduction

Wound healing is a programmed physiological process comprising four sequential phases: hemostasis, inflammation, proliferation, and tissue remodeling. Under normal conditions, acute wounds complete this process within two to six weeks. Chronic wounds, however, remain stalled in the inflammatory phase, with elevated levels of pro-inflammatory cytokines, proteases, reactive oxygen species (ROS), and senescent cells, collectively impairing tissue repair. The most prevalent types include diabetic foot ulcers, venous leg ulcers, and pressure ulcers, all of which impose a substantial burden on healthcare systems worldwide [1].

The inflammatory phase is characterized by the predominance of phagocytic cells: neutrophils, which release ROS and proteases to prevent microbial contamination, and macrophages, which secrete growth factors and cytokines that recruit fibroblasts, endothelial cells, and keratinocytes to repair damaged blood vessels. The proliferation phase involves tissue granulation, angiogenesis, and epithelialization. Finally, the remodeling phase, which may last years, replaces the provisional matrix with organized collagen bundles [1]. Patients with chronic wounds experience reduced quality of life due to persistent pain, psychological distress, and the high financial cost of treatment [2,3]. The global wound care market was projected to reach USD 7.1 billion in 2019 [4], and recent industry estimates forecast growth of at least 11% per year through 2029 [5].

The development of bioactive formulations that promote rapid and complete healing of chronic wounds is, therefore, a scientific and clinical priority. Among the most extensively studied natural compounds, curcumin [(1E,6E)-1,7-bis(4-hydroxy-3-methoxyphenyl)hepta-1,6-diene-3,5-dione] and resveratrol (3,5,4′-trihydroxystilbene) stand out for their complementary mechanisms of action. The selection of these two polyphenols for the present study was based on their well-documented antioxidant and anti-inflammatory activities, their capacity to stimulate collagen synthesis, and the synergistic effects reported in the literature for wound healing applications [6,7,8,9]. Both compounds inhibit the NF-κB signaling pathway and reduce pro-inflammatory mediators, while curcumin promotes collagen deposition and resveratrol enhances angiogenesis, constituting a complementary pharmacological profile relevant to the treatment of chronic wounds [6,10,11]. Curcumin, the principal pigment of *Curcuma longa* L. rhizomes, has a topological polar surface area of 93.1 Å^2^ and is practically insoluble in water. This physicochemical property constitutes the central challenge for its incorporation into aqueous polymeric systems: curcumin’s insolubility impairs its uniform dispersion within hydrophilic matrices, compromises the structural integrity of polymer films, and limits its diffusion into aqueous receptor media during in vitro release assays [12,13]. Therefore, the experimental difficulties observed with curcumin in the present study were anticipated based on its well-characterized biopharmaceutical profile, and the results should be interpreted in that context rather than as unexpected findings. These biopharmaceutical characteristics also justify two methodological adaptations adopted in our study, as detailed in the following sections. Resveratrol, a plant polyphenol found in high concentrations in red grapes, exists as cis-(Z) and trans-(E) isomers—the trans-form being more stable—with a water solubility of 3 mg/100 mL and UV absorption maxima at 218, 307, and 321 nm [14].

Both compounds inhibit the NF-κB signaling pathway, which is associated with prolonged inflammation, reduced angiogenesis, and impaired cell proliferation [6]. Their synergistic effects have been reported in cancer biology, hypertension, and inflammatory conditions through complementary mechanisms targeting multiple molecular pathways [15,16,17,18]. In fibroblast and keratinocyte cultures, both bioflavonoids stimulated cell division rates and scratch-wound closure without significant cytotoxicity [7]. In vivo, curcumin administered for 14 days in diabetic rats reduced NF-κB, TNF-α, and IL-6, while increasing re-epithelialization, wound closure, collagen deposition, and angiogenesis [10]; resveratrol produced analogous effects in a parallel model [11]. Both compounds also significantly increased collagen synthesis in the skin of healthy rats after chemical peeling [8], and in burn wounds, curcumin and resveratrol loaded in nanogels demonstrated in vivo healing efficacy [9]. It should be noted, however, that the pharmacological synergism described in the literature refers to dissolved or nanoencapsulated forms of these compounds.

The secretome of mesenchymal stem cells (MSCs) constitutes a third promising component for wound-healing formulations. MSCs are multipotent adult stem cells found in bone marrow, adipose tissue, umbilical cord, and dental pulp, capable of differentiating into connective, skeletal muscle, and vascular tissues [19]. Their secretome exhibits pro-angiogenic, anti-fibrotic, anti-apoptotic, anti-inflammatory, and immunomodulatory properties [20]. Secretomes can accelerate wound closure and improve the quality of healing in rats [21]; topical application accelerated wound closure in diabetic swine [22]; and its senolytic action promoted vascularization and reduced inflammation in diabetic wound beds [23]. Specifically, the secretome derived from human deciduous tooth pulp-derived MSCs (hDP-MSCs) has been characterized and validated by Payão et al. [24], who demonstrated that this hDP-MSC secretome significantly promoted keratinocyte (HaCaT) migration and in vitro wound closure under both normal and high-glucose conditions, without compromising cell viability. Furthermore, it modulated the expression of key genes involved in inflammation and tissue regeneration, including IL-1β, TNF-α, TGF-β1, and VEGF-α, in a time-dependent manner, and reduced TNF-α expression under lipopolysaccharide-induced inflammatory conditions, supporting its anti-inflammatory potential [24]. These findings position the hDP-MSC secretome as a biologically validated, cell-free therapeutic component with direct relevance to the formulation strategy.

For the delivery of these active ingredients, polymeric film dressings and hydrogels have been widely explored. Films based on poly(vinyl alcohol) (PVA) [25,26], chitosan [27], sodium alginate [28], and crosslinked hydrogels [29], as well as acrylate copolymer-based hydrogels [30], have been developed with properties including biocompatibility, exudate control, and sustained active ingredient release. These platforms aim to overcome the poor bioavailability of compounds such as curcumin, which is chemically unstable, poorly soluble in water, and rapidly metabolized.

The present work integrates two complementary undergraduate research projects evaluating the in vitro release of curcumin and resveratrol from two distinct polymeric systems: PVA/alginate/CMC films (Study 1) and an acrylate copolymer-based hydrogel (Study 2). In both cases, the formulation design includes future incorporation of MSC secretome, constituting an innovative multi-component approach with synergistic potential for the treatment of chronic wounds.

## 2. Results and Discussion

### 2.1. Interference Analysis Results

Spectral scanning confirmed the selectivity of the UV-Vis method for the individual quantification of curcumin and resveratrol in both studies. Glycerin, silicone oil, PVA, sodium alginate, CMC, and acrylate copolymer showed no significant absorption at the analytical wavelengths and did not interfere with active ingredient quantification.

### 2.2. Study 1—Evaluation of Polymeric Films

Formulations containing curcumin alone (A, and earlier developmental formulations) exhibited significant physicochemical problems: bubble formation, low mechanical resistance, high fragility, excessive adhesiveness, and rapid disintegration in aqueous medium, making release assays unfeasible. In contrast, resveratrol-only formulations (C, E, F) demonstrated greater structural stability. Formulation B was discarded due to fungal contamination after prolonged storage. Formulations C and D, containing both compounds, maintained integrity in aqueous medium but displayed irregular surface texture. Table 1 presents a consolidated summary of stability and release performance.

### 2.3. Study 1—Release Profiles

**Figure 1:**
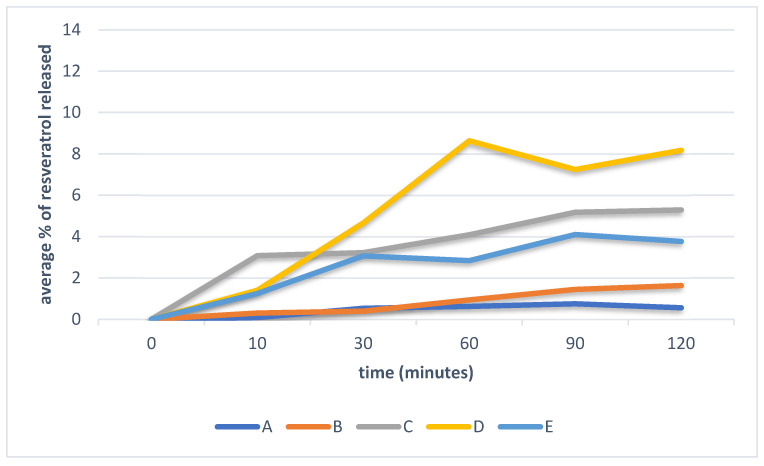
Percentage of resveratrol released vs. time for film formulations. A: resveratrol isolated (2%), no membrane; B: resveratrol isolated (2%), with membrane; C: resveratrol (2%) + curcumin (2%), no membrane; D: resveratrol (2%) + curcumin (2%), no membrane, with polysorbate 80; E: resveratrol (2%), no membrane. Source: authors (2026).

Release assay results are presented in Figure 1, where resveratrol release was evaluated under several conditions, with and without membrane and polysorbate 80. It was observed that the film formulation containing curcumin and resveratrol, evaluated in the membrane-free apparatus with polysorbate 80, provided the highest resveratrol release. Photographs of the film formulations are presented in the Supplementary Material (Figures S1 and S2).

### 2.4. Study 2—Hydrogel Physical Characterization

The acrylate copolymer-based hydrogel formulation presented macroscopic characteristics consistent with a semi-solid gel state. Apparent viscosity measured with a Brookfield viscometer (model RVDV-II+, Brookfield Engineering, Middleboro, MA, USA; spindle RV-05; 5 rpm; 25 °C) confirmed the semi-solid nature of the formulation. The gel character was additionally confirmed by visual inspection: the formulation retained its shape when transferred to an inverted container and showed no flow under gravity at room temperature, consistent with gel behavior as previously described for Aristoflex AVC-based systems [30].

### 2.5. Study 2—Release Profiles in Hydrogel

**Figure 2:**
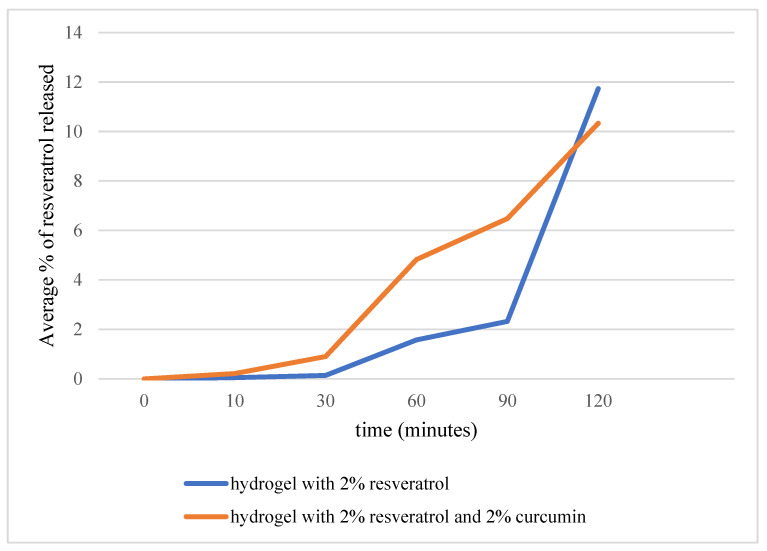
Percentage of resveratrol released from hydrogel via Franz diffusion cell (PBS pH 6.5; 35.5 ± 1 °C).

The complete release assay results for Study 2 are presented in Figure 2. Resveratrol demonstrated progressive and consistent release in the control formulation, with measurements performed across multiple independent apparatuses at each time point (n = 3–4 at t = 60–90 min; n = 9 at t = 10 min), confirming the reproducibility of the release profile. Curcumin absorbance values were below the lower limit of linearity of the calibration curve (25 μg/mL) in all time points and in all replicates (n = 3–5 per time point), rendering quantitative results unreliable. The consistency of these sub-limit readings across independent measurements confirms that the absence of quantifiable curcumin release reflects a genuine physicochemical limitation rather than experimental error. Photographs of the hydrogel formulations are presented in the Supplementary Material (Figures S3 and S4).

The results obtained in both studies converge: resveratrol demonstrated a sustained, reproducible in vitro release profile across both evaluated polymeric platforms, whereas curcumin exhibited unsatisfactory release, primarily due to its low aqueous solubility.

Confirmation of UV-Vis method selectivity by spectral scanning was an essential methodological step. Spectral overlap is one of the main challenges of spectrophotometric methods applied to complex mixtures [31], and verifying that no excipient interfered with the quantification of the active ingredient ensured the reliability of subsequent release assay results. With curcumin at 424.9 nm and resveratrol at 305.0 nm, and no excipient absorbing significantly at either wavelength, the method proved adequate for individual quantification of both compounds.

In Study 1, curcumin-only films showed behavior consistent with the literature: the addition of curcumin to polymeric matrices reduced tensile strength and maximum elongation, indicating lower structural cohesion [12,13], which manifested as high fragility, excessive adhesiveness, and rapid disintegration in PBS.

The selection of the ternary PVA/alginate/CMC system for Study 1 was based on the complementary functional contributions of each component: PVA acts as the primary film-forming agent, providing mechanical resistance and film continuity [25,26]; sodium alginate contributes to exudate absorption capacity and biocompatibility, a relevant property for wound dressings in direct contact with wound fluid [28]; and CMC functions as a suspending agent and viscosity modulator, improving polymer matrix homogeneity [29]. This combination has been employed in wound dressing formulations seeking a balance between structural integrity, exudate management, and active ingredient incorporation [29]. For Study 2, the acrylate copolymer (Ammonium Acryloyldimethyltaurate/VP Copolymer) was chosen as the hydrogel-forming agent due to its capacity to form stable gels at low concentrations without requiring pH adjustment, its compatibility with hydrophobic active ingredients, and its documented use in topical pharmaceutical formulations [30].

In Study 2, resveratrol incorporated into the acrylate copolymer hydrogel exhibited gradual, progressive release in both isolated and combined formulations. This behavior is attributed to resveratrol’s hydrophobic nature: when embedded in the polymeric network, release is governed by diffusion through the matrix, avoiding a burst release and favoring maintenance of therapeutic concentrations over time. This characteristic is highly desirable for chronic wound management, where sustained antioxidant and anti-inflammatory activity is required. The diffusion-controlled release behavior observed for resveratrol is consistent with findings reported by Pushpalatha et al. [32], who described progressive, near-linear resveratrol release from hydrogel systems over comparable time intervals, attributing the profile to Fickian diffusion through the polymeric network. Comparable sustained-release patterns have also been reported for resveratrol incorporated in PVA-based films and cyclodextrin hydrogels, in which release rates were modulated by polymer crosslinking density and active ingredient–polymer interactions [32]. The resveratrol release profile observed in the present study, approximately 10–15% over 120 min, is within the range described for analogous polymeric systems, reinforcing the plausibility of diffusion as the dominant release mechanism. Quantitative kinetic modeling (e.g., Higuchi, Korsmeyer-Peppas) was not performed due to the limited number of time points and the exploratory character of the study; this analysis is identified as a priority for future investigations with a larger dataset.

Curcumin results were unsatisfactory in both systems. Despite imparting its characteristic yellow-orange color to the receptor medium, its absorbance values were systematically outside the analytical method’s linear range, preventing reliable quantification. Comparative studies have shown that hydrogel-based systems tend to retain curcumin within the matrix, leading to lower release rates than nanoparticle systems [13]. Nanoencapsulation strategies, particularly liposomes [33] and polymeric nanoparticles [10], have demonstrated improved curcumin solubility, stability, and bioavailability. Reformulation of curcumin using nanotechnology, therefore, represents the logical next step to enable its incorporation into the proposed bioactive wound dressing.

The MSC secretome component was not quantitatively evaluated in the present work due to the high cost of cytokine and growth factor assay kits. However, its biological activity has been validated by our research group: Payão et al. [24] demonstrated that the same hDP-MSC secretome used in the present Formulation significantly promoted keratinocyte migration and in vitro wound closure in scratch assays, modulated key regenerative genes (IL-1β, TNF-α, TGF-β1, VEGF-α) in a time-dependent manner, and reduced TNF-α expression under inflammatory conditions induced by lipopolysaccharide, confirming its anti-inflammatory potential. These findings provide direct biological validation for the secretome component of Study 2. Secretome components are predominantly water-soluble, which would favor their release into the aqueous wound environment. The combination of resveratrol, with proven antioxidant, anti-inflammatory, and pro-angiogenic activity [6,8], and the validated hDP-MSC secretome, delivered in a single polymeric platform, represents an innovative and potentially synergistic cell-free strategy for chronic wound management.

This study has limitations inherent to its exploratory and preliminary character. The adapted release apparatus and variations in film-drying conditions may have introduced uncontrolled variability. The number of independent replicates varied across time points and conditions, ranging from 2 to 9. At the same time, this enabled descriptive statistics (mean ± SD), variable replication, and formal inferential comparisons between groups. Furthermore, all experiments were conducted in vitro using PBS as the receptor medium, which only partially simulates the complex chronic wound microenvironment (enzymes, variable pH, plasma proteins, and possible biofilm). The clinical relevance of the observed release profiles remains to be confirmed in cellular and preclinical in vivo models.

## 3. Conclusions

Both studies converge to demonstrate that resveratrol at 2% (*w*/*w*) exhibits progressive, sustained, and reproducible in vitro release profiles from PVA/alginate/CMC polymeric films and an acrylate copolymer-based hydrogel, supporting the viability of these matrices as sustained-release platforms for bioactive wound dressings. Formulations E and F (Study 1) and the resveratrol control (Study 2) are recommended for continuation as the most suitable for further development.

Curcumin demonstrated unsatisfactory release in both platforms, attributed to its low aqueous solubility. Nanoencapsulation strategies, particularly liposomes and polymeric nanoparticles, are recommended to overcome this limitation and enable their inclusion in the proposed Formulation.

The association of resveratrol with the biologically validated hDP-MSC secretome (Payão et al. [24]) within a single polymeric platform represents an innovative multi-component approach with high therapeutic potential for chronic wound healing, warranting further investigation in cellular and in vivo preclinical models, including quantitative profiling of secretome release.

## 4. Materials and Methods

### 4.1. Study Design

Two in vitro experimental studies were conducted. Study 1 evaluated polymeric films, and Study 2 evaluated an acrylate copolymer-based hydrogel.

### 4.2. Study 1—Polymeric Films

#### 4.2.1. Formulation Preparation

All materials were of pharmaceutical grade and were used as received, without any pre-treatment. PVA (MW ~ 72,000 g/mol; degree of hydrolysis ≥ 99%; Synth, São Paulo, Brazil), sodium alginate (medium viscosity; Synth, São Paulo, Brazil), CMC (MW ~ 250,000 g/mol; Synth, São Paulo, Brazil), glycerin (purity ≥ 99.5%; Synth, São Paulo, Brazil), silicone oil (pharmaceutical grade; Synth, São Paulo, Brazil), methylparaben (purity ≥ 99%; Synth, São Paulo, Brazil), curcumin (purity ≥ 95%; Florien, São Paulo, Brazil), resveratrol (trans-form; purity ≥ 99%; Florien, São Paulo, Brazil), and deionized water were used. Polymeric films were prepared incorporating curcumin and/or resveratrol at 2% (*w*/*w*). A 15% (*w*/*v*) PVA stock solution was prepared under controlled heating (60 °C—Prolab magnetic stirrer model PRO-2000HA, Prolab, São Paulo, Brazil) with constant magnetic stirring until complete dissolution. CMC was hydrated in a water bath at 40 °C for 1 h (SolidSteel ultrasonic bath with heating, model SSBu 10L, Piracicaba, Brazil). Sodium alginate was dissolved under heating below 50 °C. Active ingredients were dispersed in glycerin and added to the PVA solution. After homogenization of all components, the pH was adjusted to 5.8 with 10% (*w*/*v*) acetic acid. pH was measured using a calibrated digital pH meter (Digimeto DM-20, Digimed, São Paulo, Brazil). Formulations were weighed on an analytical balance (Marte AY220, Marte Científica, São Paulo, Brazil; readability 0.0001 g). Formulations were poured into circular silicone molds, dried in an oven at 50 °C (Fanen Model 315SE forced-air oven, Fanen, São Paulo, Brazil) and subsequently stored in a desiccator. The compositions of the main evaluated formulations are presented in Table 2.

#### 4.2.2. Release Assays—Study 1

In vitro release profiles were evaluated using phosphate-buffered saline (PBS, pH 6.5; 35.5 ± 1 °C) as the receptor medium, which partially simulates the wound microenvironment [32,34]. Films were positioned either on semi-permeable cellulose acetate membranes (diameter 47 mm; porosity ~ 4500 Å) pre-soaked in the receptor medium, or directly immersed in the receptor medium, in 80 mL hermetic flasks. The choice of methodology was formulation-dependent: films were initially positioned on the membrane according to standard Franz cell procedures; however, this configuration proved inadequate for certain formulations due to their physical characteristics (excessive adhesiveness and limited mechanical resistance), which prevented uniform contact with the membrane and compromised reproducibility. Direct immersion of these films in the receptor medium was therefore adopted as an adapted approach to enable comparative evaluation of release behavior, a strategy that has been reported as acceptable for films with poor mechanical integrity when the primary objective is to characterize the kinetics of active ingredient diffusion into an aqueous medium [32,34]. Aliquots were collected at 10, 30, 60, 90, and 120 min for UV-Vis spectrophotometric quantification. For formulation C, additional assays were performed with polysorbate 80 added to the receptor medium, as a surfactant widely employed to improve curcumin aqueous solubilization in release studies [32]. A comparison between assays conducted on semi-permeable membranes and those performed by direct immersion was therefore performed, given that the mechanical fragility of curcumin-containing films precluded uniform membrane contact in standard Franz cell configurations.

### 4.3. Study 2—Hydrogel

#### 4.3.1. Formulation

The test formulation was a hydrogel of simplified composition, developed with a focus on formulation simplicity and excipient safety for application to chronic wounds. The final composition comprised acrylate copolymer (Aristoflex AVC; INCI: Ammonium Acryloyldimethyltaurate/VP Copolymer; Clariant, München, Germany) (1.8%), preservative (0.8%), emulsifying agent (0.2%), curcumin (2%), resveratrol (2%), MSC secretome (0.2%), and purified water to 100%. Aristoflex AVC is an anionic synthetic polymer widely used in topical pharmaceutical and cosmetic formulations, capable of forming transparent, stable hydrogels at concentrations of 1–3% (*w*/*w*) without requiring pH adjustment, and presenting compatibility with electrolytes and hydrophobic active ingredients [30]. The gel state of the prepared formulation was confirmed by apparent viscosity measurement and by visual inspection, as described in Section 4.4.

The hydrogel was prepared by dispersing the acrylate copolymer in purified water under constant magnetic stirring until complete homogenization. Next, the preservative and emulsifier were incorporated, followed by curcumin, resveratrol, and MSC secretome. This secretome was obtained from MSCs derived from deciduous tooth pulp, collected from pediatric patients, as described by Payão et al. [24]. The secretome was used as the conditioned medium fraction collected after 48 h of culture of hDP-MSCs in serum-free DMEM, centrifuged at 300× *g* for 5 min to remove cell debris, and stored at −80 °C until use, following the protocol described by Payão et al. [24]. This conditioned medium contains soluble paracrine factors, including growth factors, cytokines, and extracellular vesicles, collectively referred to as the MSC secretome. For release assays, control formulations were also prepared: placebo, curcumin-only control, resveratrol-only control, and curcumin + resveratrol combined (without secretome).

#### 4.3.2. Release Assays—Study 2

Prior to the final release assay, a pilot experiment was conducted using 1.0 g of gel per apparatus to verify the methodology’s feasibility and identify conditions requiring adjustment. Based on these preliminary results, the applied mass was reduced to 0.5 g per apparatus to achieve a more appropriate release profile. The final assay was conducted with 0.5 g of each Formulation uniformly distributed over cellulose acetate semi-permeable membranes (47 mm, 4500 Å, Kasvi, São José dos Pinhais, Brazil), with 74.5 mL of PBS (pH 6.5) as the receptor medium, maintained at 35.5 ± 1 °C. Individual apparatuses were assembled for each collection time point (10, 30, 60, 90, and 120 min) and maintained in a thermostated ultrasonic bath at 35.5 ± 1 °C throughout the assay. Although the standard Franz diffusion cell protocol involves sequential aliquot withdrawal from a single cell with volume replacement, the individual apparatus approach was adopted to avoid the need for sink condition corrections and volume replacement calculations. To minimize inter-apparatus variability, measurements were performed in triplicate or higher (n = 3 at t = 60 and 90 min; n = 4 at t = 90 min; n = 9 at t = 10 min), and results are expressed as means ± standard deviations as presented in Table S2 (Supplementary Material). At each time point, a 3 mL aliquot of the receptor medium was collected for quantification of released curcumin and/or resveratrol by UV-Vis spectrophotometry (UV-Vis Varian Spectrophotometer, Palo Alto, CA, USA) at 425 nm and 305 nm, respectively.

#### 4.3.3. Calibration Curves

For curcumin quantification, stock solutions were prepared in PBS (pH 6.5) with a few drops of Tween 80 to promote aqueous dissolution. Aliquots were diluted in PBS (pH 6.5) to yield concentrations of 25, 37.5, 50, 62.5, and 75 µg/mL. For resveratrol, stock solutions were prepared in PBS (pH 6.5), and final concentrations were: 1, 2, 3, 4, 5, and 6 µg/mL. Linear regression analysis yielded the following equations: for curcumin, concentration (µg/mL) = (absorbance + 0.09826)/0.01141; for resveratrol, Concentration (µg/mL) = (Absorbance − 0.02701)/0.11808. Correlation coefficients (r^2^) exceeded 0.999 for both compounds, confirming excellent linearity within the evaluated concentration ranges. The calibration curves are presented in the Supplementary Material (Figures S5 and S6).

### 4.4. Interference Analysis

In both studies, individual spectral scans of each formulation component were performed over 200–800 nm to confirm the absence of overlapping absorbance bands that could interfere with active ingredient quantification [31]. This step was essential to establish the selectivity of the UV-Vis method prior to release assays.

### 4.5. Statistical Analysis

Results were organized in tables and figures. Descriptive statistical analyses (means and standard deviations) were performed using Microsoft Excel^®^ Version 2606 Build 16.0.20131.20112) 64-bit (Microsoft Corporation, Redmond, WA, USA). The number of independent replicates (n) varied by time point and Formulation as detailed in the Supplementary Material (Tables S2 and S3); standard deviations were calculated using n − 1 in the denominator. Given the exploratory nature of the study and the variable number of replicates across time points, inferential statistical comparisons between groups were not performed.

## Figures and Tables

**Table 1:** Summary of stability and release profile of polymeric film formulations (Study 1).

Active Ingredient	Stability	Release Profile	Main Observations
Curcumin	Low	Irregular	Early disintegration; low solubility; unfeasible for release assay
Resveratrol	High	Sustained	Progressive, stable, reproducible release
Curcumin + Resveratrol	Moderate	Unstable	Curcumin compromises the matrix and reduces resveratrol release efficiency

**Table 2:** Composition of the main polymeric film formulations evaluated in Study 1.

Component	A (C)	B (R)	C (C + R)	D (C + R)	E (R)	F (C + R)
PVA stock solution	26.7 g	26.7 g	22.0 g	22.0 g	22.0 g	22.0 g
Sodium alginate	0.60 g	0.60 g	0.60 g	0.60 g	0.60 g	0.60 g
CMC	0.15 g	0.15 g	0.15 g	0.15 g	0.15 g	0.15 g
Curcumin	2 g	-	2 g	0.67 g	-	0.67 g
Resveratrol	-	2 g	2 g	2 g	2 g	2 g
Glycerin	10 mL	10 mL	15 mL	15 mL	15 mL	15 mL
Silicone oil	2 drops	2 drops	2 drops	2 drops	2 drops	2 drops
Methylparaben	0.15 g	0.15 g	0.15 g	0.15 g	0.15 g	0.15 g
Deionized water	q.s. 100 g	q.s. 100 g	q.s. 100 g	q.s. 100 g	q.s. 100 g	q.s. 100 g

CMC: carboxymethylcellulose; (C): with curcumin; (R): with resveratrol; (C + R): with curcumin and resveratrol; PVA: poly(vinyl alcohol); q.s.: quantum satis.

## Data Availability

The original contributions presented in this study are included in the article. Further inquiries can be directed to the corresponding author.

## Supplementary Materials

The following supporting information can be downloaded at: https://www.mdpi.com/article/10.3390/gels12070653/s1, Figure S1. Film formulation with curcumin 2% (*w*/*w*). Photographs show film removal from mold and general aspect. Source: authors (2025). Figure S2. Film formulation with curcumin 2% (*w*/*w*) and resveratrol 2% (*w*/*w*). Dark orange-brown coloration characteristic of high curcumin content. Source: authors (2025). Figure S3. Hydrogel formulations used in Study 2 in vitro release assays. (A) Placebo; (B) curcumin-containing formulation; (C) curcumin + resveratrol combined formulation. Source: authors (2025). Figure S4. Resveratrol-containing hydrogel formulation after five months of storage at room temperature, protected from light and heat. Source: authors (2025). Figure S5. Calibration curve of curcumin in PBS pH 6.5 (with Tween 80). Concentration range: 25–75 µg/mL; λ = 424.9 nm. Linear equation: Concentration (µg/mL) = (Absorbance + 0.09826)/0.01141; r^2^ = 0.999. Figure S6. Calibration curve of resveratrol in PBS pH 6.5. Concentration range: 1–6 µg/mL; λ = 305.0 nm. Linear equation: Concentration (µg/mL) = (Absorbance − 0.02701)/0.11808; r^2^ = 0.9992. Table S1. Resveratrol release data from hydrogel formulations (preliminary experiment; 1.0 g gel). Note: this table presents results from the pilot experiment, which was conducted prior to the protocol adjustment described in Section 4.3.2 of the manuscript. The validated final data (0.5 g gel) are presented in Tables S2 and S2 (cont.) below. Table S2. Individual absorbance values for resveratrol released from the acrylate copolymer-based hydrogel: Study 2 (Franz diffusion cell; 0.5 g gel; PBS pH 6.5; 35.5 ± 1 °C). Table S3. Individual absorbance values for resveratrol and curcumin released from PVA/alginate/CMC polymeric films: Study 1 (PBS pH 6.5; 35.5 ± 1 °C).

## References

[1] Frykberg R.G., Banks J. (2015). Challenges in the treatment of chronic wounds. Adv. Wound Care.

[2] Vogt T.N., Koller F.J., Santos P.N.D., Lenhani B.E., Guimarães P.R.B., Kalinke L.P. (2020). Quality of life assessment in chronic wound patients using the Wound-QoL and FLQA-Wk instruments. Investig. Educ. Enferm..

[3] Oliveira A.C., Rocha D.M., Bezerra S.M.G., Andrade E.M.L.R., Santos A.M.R., Nogueira L.T. (2019). Quality of life of people with chronic wounds. Acta Paul. Enferm..

[4] Gupta A., Kowalczuk M., Holton J., Malhotra A., Baguneid M., Saeed T., Bhaskaran N., Dittrich M. (2019). The production and application of hydrogels for wound management: A review. Eur. Polym. J..

[5] Mordor Intelligence (2025). Bioactive Wound Dressing Market Size & Share Analysis—Growth Trends & Forecasts (2025–2030).

[6] Yadav J.P., Singh A.K., Garg A., Bansal Y., Bansal G., Kang W., Medhi B. (2024). Phytoconstituents as modulators of NF-κB signaling: Investigating therapeutic potential for diabetic wound healing. Biomed. Pharmacother..

[7] Jagiełło K., Uchańska O., Matyja K., Jackowski M., Wiatrak B., Kubasiewicz-Ross P., Karuga-Kuźniewska E. (2023). Supporting the wound healing process—Curcumin, resveratrol and baicalin in in vitro wound healing studies. Pharmaceuticals.

[8] Gonçalves G.M.S., Schaffazick S.R., Cruz L., Pohlmann A.R., Guterres S.S. (2017). Formulations containing curcumin or trans-resveratrol increase dermal thickness in rats submitted to chemical peeling. J. Cosmet. Dermatol. Sci. Appl..

[9] Alyoussef A., Al-Gayyar M., Makhdoom R., Almaeen A. (2021). The beneficial activity of curcumin and resveratrol loaded in nanoemulgel for healing of burn-induced wounds. J. Drug Deliv. Sci. Technol..

[10] Li F., Liu X., Tong Z., Liu C., Liu X. (2019). Curcumin-loaded chitosan nanoparticles promote diabetic wound healing via attenuating inflammation in a diabetic rat model. J. Biomater. Appl..

[11] Youjun D., Huang Y., Lai Y., Ma Z., Wang X., Chen B., Tan Q. (2023). Mechanisms of resveratrol against diabetic wound by network pharmacology and experimental validation. Ann. Med..

[12] Carvalho R.A., Grosso C.R.F. (2004). Characterization of gelatin based films modified with transglutaminase, glyoxal and formaldehyde. Food Hydrocoll..

[13] Gualmatán E.A.C. (2025). Hidrogeles Inyectables con Curcumina con Potencial Aplicación en el Tratamiento de Cáncer de Mama. Bachelor’s Thesis.

[14] Kim S., Chen J., Cheng T., Gindulyte A., He J., He S., Li Q., Shoemaker B.A., Thiessen P.A., Yu B. (2023). PubChem 2023 update. Nucleic Acids Res..

[15] Majumdar A.P.N., Banerjee S., Nautiyal J., Patel B.B., Patel V., Du J., Yu Y., Elliott A.A., Levi E., Sarkar F.H. (2009). Curcumin synergizes with resveratrol to inhibit colon cancer. Nutr. Cancer.

[16] Jaisamut P., Wiwattanawongsa K., Graidist P., Sangwanit K., Wiwattanapatapee R. (2018). Enhanced oral bioavailability of curcumin using a supersaturatable self-microemulsifying system incorporating a hydrophilic polymer. AAPS PharmSciTech.

[17] Patra S., Pradhan B., Nayak R., Behera C., Das S., Nayak P.K. (2021). Chemotherapeutic efficacy of curcumin and resveratrol against cancer: Chemoprevention, chemoprotection, drug synergism and clinical pharmacokinetics. Semin. Cancer Biol..

[18] Ghaeini Hesarooeyeh Z., Rafati Rahimzadeh M., Seyedalipour B. (2024). Effect of resveratrol and curcumin and the potential synergism on hypertension: A mini-review of human and animal model studies. Phytother. Res..

[19] Pittenger M.F., Discher D.E., Péault B.M., Phinney D.G., Hare J.M., Caplan A.I. (2019). Mesenchymal stem cell perspective: Cell biology to clinical progress. npj Regen. Med..

[20] Daneshmandi L., Shah S., Bhatt R., Laurencin C.T. (2020). Emergence of the stem cell secretome in regenerative engineering. Trends Biotechnol..

[21] Sun J., Zhang Y., Song X., Zhu J., Zhu Q. (2019). The healing effects of conditioned medium derived from mesenchymal stem cells on radiation-induced skin wounds in rats. Cell Transplant..

[22] Irons R.F., Cahill K.W., Rattigan D.A., Marcotte J.H., Vasquez M.R., Driscoll A.T., Garza J.R. (2018). Acceleration of diabetic wound healing with adipose-derived stem cells, endothelial-differentiated stem cells, and topical conditioned medium therapy in a swine model. J. Vasc. Surg..

[23] Wang B., Tchkonia T., Kirkland J.L., Wan Y. (2023). Human fetal mesenchymal stem cells secretome promotes scarless diabetic wound healing through heat-shock protein family. Bioeng. Transl. Med..

[24] Payão T.S., Pellegrini V., Morari J., Gonçalves G.M.S., de Godoy M.C.X., Gambero A., Reis L.O., Velloso L.A., Araújo E.P., Pascoal L.B. (2025). The secretome of human deciduous tooth-derived mesenchymal stem cells enhances in vitro wound healing and modulates inflammation. Pharmaceutics.

[25] Hedayatyanfard K., Hosseinzadeh-Attar M.J., Zare Mehrjardi M., Taheri S., Basiri A., Bailey C.J., Habibi M., Esmaeili N. (2019). Semi-IPN films and electrospun nanofibers based on chitosan/PVA as an antibacterial wound dressing. Iran. J. Pharm. Res..

[26] Nour S., Imani R., Sharifi A.M. (2022). Angiogenic effect of a nanoniosomal deferoxamine-loaded poly(vinyl alcohol)–egg white film as a promising wound dressing. ACS Biomater. Sci. Eng..

[27] Mehmood Y., Shahid H., Arshad N., Rasul A., Jamshaid T., Jamshaid M., Jamshaid U., Uddin M.N., Kazi M. (2023). Amikacin-loaded chitosan hydrogel film cross-linked with folic acid for wound healing application. Gels.

[28] Tan W.S., Arulselvan P., Ng S.F. (2020). Healing effect of Vicenin-2 (VCN-2) on human dermal fibroblast (HDF) and development VCN-2 hydrocolloid film based on alginate as potential wound dressing. Biomed. Res. Int..

[29] Ávila-Salas F., Marican A., Pinochet S., Carreño G., Valdés O., Venegas B., Donoso W., Cabrera-Barjas G., Vijayakumar S., Durán-Lara E.F. (2019). Film dressings based on hydrogels: Simultaneous and sustained-release of bioactive compounds with wound healing properties. Pharmaceutics.

[30] Jin S.G., Yousaf A.M., Kim K.S., Kim D.W., Kim D.S., Li Z., Youn Y.S., Han J.H., Cho K.H., Choi H.G. (2016). Influence of hydrophilic polymers on functional properties and wound healing efficacy of hydrocolloid based wound dressings. Int. J. Pharm..

[31] Paschoal L.R., Ferreira W.A., Prado M.R.D., Vilela A.P.O. (2003). Aplicação do método da espectrofotometria de derivadas na identificação e doseamento simultâneo de sistemas multicomponentes. Rev. Bras. Ciênc. Farm..

[32] Pushpalatha R., Selvamuthukumar S., Kilimozhi D. (2019). Cyclodextrin nanosponge based hydrogel for the transdermal co-delivery of curcumin and resveratrol: Development, optimization, in vitro and ex vivo evaluation. J. Drug Deliv. Sci. Technol..

[33] Santana T.F. (2021). Análise da Inflamação e Estresse Oxidativo no Processo de Cicatrização Tecidual Após o Uso Combinado de Lipossomas com Curcumina. Master’s Thesis.

[34] Afzal S., Mehwish K., Afzal F., Lim C.W., Kim B. (2023). Formulation and characterization of polymeric cross-linked hydrogel patches for topical delivery of antibiotic for healing wound infections. Polymers.

