# Supplementary material for: In Vitro Release of Curcumin and Resveratrol from Polymeric Systems: Films and Hydrogel
Source: Gels. 2026 Jul 21;12(7):653. doi: 10.3390/gels12070653 (PMC13408998; doi:10.3390/gels12070653)
Supplement: Supplementary file 1 [file gels-12-00653-s001.zip › gels-4352040-supplementary.pdf]

Supplementary Material

In Vitro Release of Curcumin and Resveratrol from Polymeric Systems: Films and Hydrogel

Section S1. Photographs of Formulations

Figure S1. Film formulation with curcumin at 2% (w/w). The sequence of photographs illustrates the film during removal from the mold, showing its characteristic yellow-orange color, flexibility, and surface homogeneity.

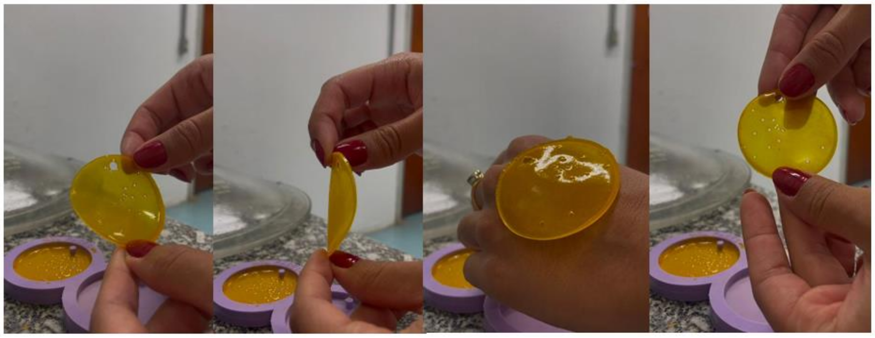

Figure S1. Film formulation with curcumin 2% (w/w). Photographs show film removal from mold and general aspect. Source: authors (2025).

Figure S2. Film formulation with curcumin 2% (w/w) and resveratrol 2% (w/w). The photograph shows the film's characteristic dark orange-brown color due to the high curcumin content.

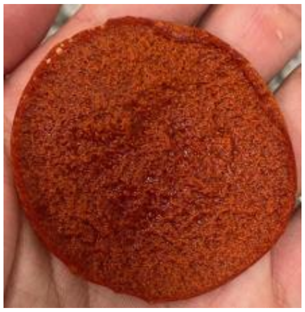

Figure S2. Film formulation with curcumin 2% (w/w) and resveratrol 2% (w/w). Dark orange-brown coloration characteristic of high curcumin content. Source: authors (2025).

Figure S3. Hydrogel formulations used in Study 2 release assays. (A) Placebo formulation; (B) curcumin-containing formulation; (C) curcumin + resveratrol combined formulation.

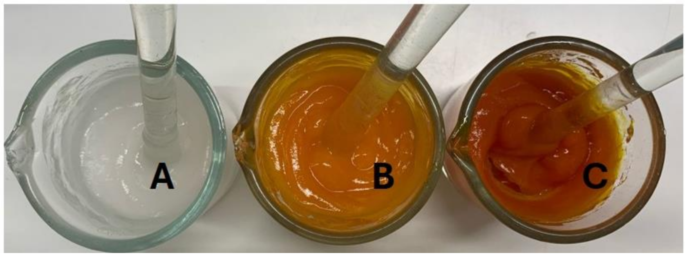

Figure S3. Hydrogel formulations used in Study 2 in vitro release assays. (A) Placebo; (B) curcumin-containing formulation; (C) curcumin + resveratrol combined formulation. Source: authors (2025).

Figure S4. Resveratrol-containing hydrogel formulation after five months of storage at room temperature, protected from light and heat. The photographs demonstrate the physical-chemical stability of the formulation under ambient storage conditions.

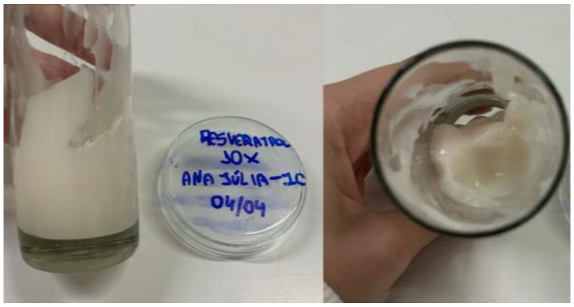

Figure S4. Resveratrol-containing hydrogel formulation after five months of storage at room temperature, protected from light and heat. Source: authors (2025).

Section S2. Analytical Calibration Curves

Calibration curves for curcumin and resveratrol were established in phosphate-buffered saline (PBS, pH 6.5) prior to both Study 1 and Study 2 release assays, using UV-Vis spectrophotometry. The same curves were applied to both studies, as both employed identical receptor medium and quantification methodology.

Figure S5. Calibration curve of curcumin in PBS pH 6.5 (with Tween 80). Concentration range: 25–75 µg/mL; λ = 424.9 nm. Linear equation: Concentration (µg/mL) = (Absorbance + 0.09826) / 0.01141; r^2 = 0.999.

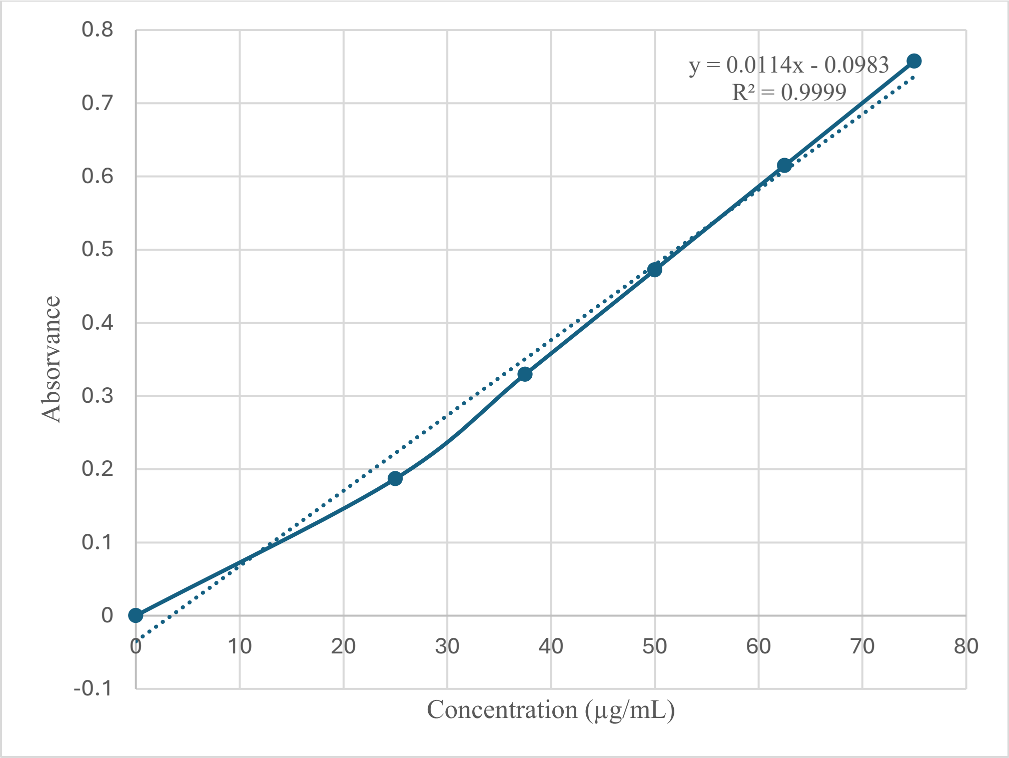

Figure S6. Calibration curve of resveratrol in PBS pH 6.5. Concentration range: 1–6 µg/mL; λ = 305.0 nm. Linear equation: Concentration (µg/mL) = (Absorbance − 0.02701) / 0.11808; r^2 = 0.9992.

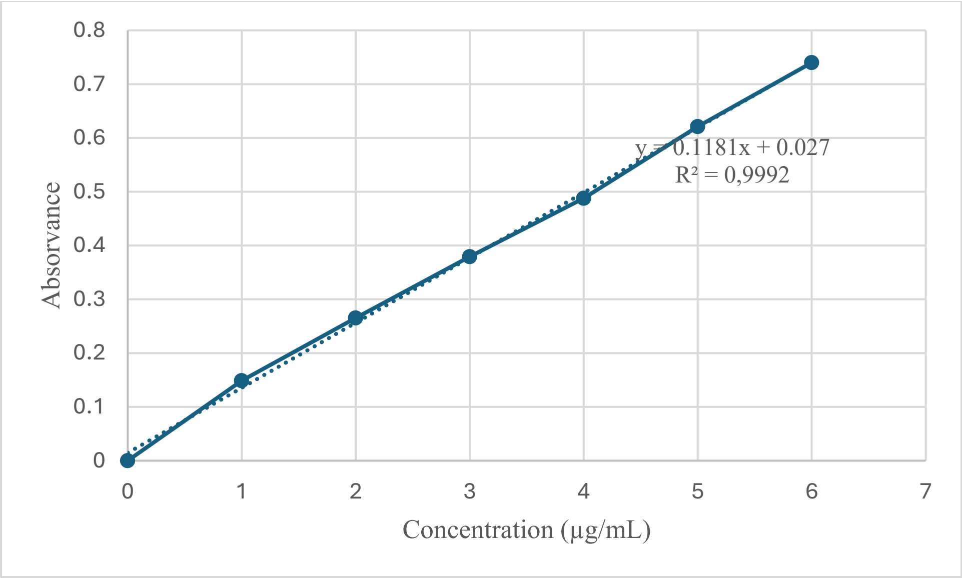

Section S3. Quantitative Release Data — Hydrogel (Study 2)

Table S1. Resveratrol release data from hydrogel formulations (preliminary experiment; 1.0 g gel). Note: this table presents results from the pilot experiment, which was conducted prior to the protocol adjustment described in section 4.3.2 of the manuscript. The validated final data (0.5 g gel) are presented in Tables S2 and S2 (cont.) below.

Hydrogel	**Time (min)**	**Concentration (µg/mL)**	**Released (%)**
Resveratrol	10	0 ± 0	0.05
	30	0.07 ± 0.00025	0.14
	60	1.92 ± 0.00038	1.57
	90	2.83 ± 0.00052	2.32
	120	14.31 ± 0.00118	11.73
Resveratrol and Curcumin (resveratrol analysis)	10	0.26 ± 0.00044	0.21
	30	1.17 ± 0.00052	0.90
	60	5.88 ± 0.00036	4.82
	90	7.92 ± 0.00066	6.48
	120	12.60 ± 0.00170	10.33

Section S4. Individual Absorbance Values — Raw Data and Descriptive Statistics

Tables S2 and S3 present the individual absorbance values (AU) measured by UV-Vis spectrophotometry for each independent replicate at each time point in both studies. Mean and standard deviation (SD) were calculated with n−1 in the denominator. Rep. = independent Franz diffusion cell apparatus (Study 2) or independent film sample (Study 1) indicates no measurement performed for that replicate.

Table S2. Individual absorbance values for resveratrol released from the acrylate copolymer-based hydrogel: Study 2 (Franz diffusion cell; 0.5 g gel; PBS pH 6.5; 35.5 ± 1 °C).

Time (min)	Compound	Rep. 1	Rep. 2	Rep. 3	Rep. 4	Rep. 5	Rep. 6	Rep. 7	Rep. 8	Rep. 9	Mean	SD
10	**Resveratrol 2% (λ = 305 nm)**	0.0198	0.0203	0.0205	0.0207	0.0213	0.0216	0.0222	0.0230	0.0229	**0.0214**	0.0011
30		0.0355	0.0350	0.0352	-	-	-	-	-	-	**0.0352**	0.0003
60		0.2538	0.2545	0.2544	-	-	-	-	-	-	**0.2542**	0.0004
90		0.3607	0.3613	0.3602	0.3602	-	-	-	-	-	**0.3606**	0.0005
120		1.7251	1.7238	1.7272	1.7271	-	-	-	-	-	**1.7258**	0.0016

t = 10 and t = 30 min: absorbance below the lower limit of linearity (1 µg/mL; Abs ≥ 0.145); release not reliably quantifiable at these time points. (a) t = 120 min: absorbance above the upper limit of linearity (6 µg/mL; Abs ≤ 0.736); value reported as measured.

Table S2 (cont.). Individual absorbance values for curcumin released from the acrylate copolymer-based hydrogel: Study 2.

Time (min)	Compound	Rep. 1	Rep. 2	Rep. 3	Rep. 4	Rep. 5	Mean	SD
10	**Curcumin 2% (λ = 425 nm)**	0.0157	0.0152	0.0156	0.0153	0.0102	**0.0144**	0.0024
30		0.0097	0.0096	0.0099	0.0097	-	**0.0097**	0.0001
60		0.0008	0.0008	0.0008	-	-	**0.0008**	0.0000
90		0.0042	0.0044	0.0049	0.0043	0.0043	**0.0044**	0.0003
120		0.0044	0.0045	0.0045	-	-	**0.0045**	0.0001

All curcumin absorbance values were below the lower limit of linearity of the calibration curve (25 µg/mL; λ = 425 nm). Quantitative release could not be reliably determined; values are presented for transparency only.

Table S3. Individual absorbance values for resveratrol and curcumin released from PVA/alginate/CMC polymeric films: Study 1 (PBS pH 6.5; 35.5 ± 1 °C).

Time (min)	Compound / Condition	Rep. 1	Rep. 2	Rep. 3	Mean	SD
10	**Resveratrol 2%, isolated film, no membrane (λ = 305 nm)**	0.1320	0.1443	-	**0.1381**	0.0087
30		0.4188	0.2881	-	**0.3535**	0.0924
60		0.2925	0.4351	0.4328	**0.3868**	0.0817
90		0.8448	0.5058	0.4779	**0.6095**	0.2043
120		0.7229	0.4806	0.4722	**0.5586**	0.1424

n = 2 at t = 10 and 30 min; n = 3 at t = 60, 90, and 120 min.

Time (min)	Compound / Condition	Rep. 1	Rep. 2	Mean	SD
30	**Curcumin 2%, isolated film, with membrane (λ = 425 nm)**	0.0261	0.0047	**0.0154**	0.0151
60		0.0089	0.0220	**0.0154**	0.0093
90		0.0818	0.0477	**0.0648**	0.0241

n = 2 at t = 30, 60, and 90 min. t = 120 min not evaluated due to film disintegration in the receptor medium.
